# 

**Published:** 1903-05-09

**Authors:** 


### NOTICE TO CORRESPONDENTS.

All MSS., Letters, Books for Review, and other matters intended for the Editor should be addressed The Editor, "The Hospital," The Hospital Buildings, 28 & 29 Southampton Street, Strand, W.C.

The Editor cannot undertake to return rejected MS., even when accompanied by stamped directed envelope.

**Notice to Advertisers and Subscribers.**

All Advertisements, Orders for Copies of The Hospital, and Business Communications should be addressed to **THE MANAGER, (Not to the Editor)**, The Hospital, 28 & 29 Southampton Street, Strand, London, W.C.

The Cost of Subscription, post free, is as follows.—Per week, 3½d.; per quarter, 3s. 9d.; per half-year, 7s. 6d.; per annum, 15s. Foreig and Colonial:—Per annum, 19s. 6d., payable, in all cases, in advance.

**NOTICE.—Vol. XXXIII. of "The Hospital" (October 1902 to March 1903) in handsome cloth binding, will shortly be on sale at the office of this Journal, price 7s. 6d. Cases for binding the Numbers contained in this Volume 1s. 6d. Index to Vol. XXXIII., 2½d., post free.**

**The monthly part for May is now ready. Price 1s., by post 1s. 3d.**

### BOOKS RECEIVED.

Cassell and Co., Ltd.
"Tropical Diseases." New Edition. By Patrick Manson.

Health Resorts Bureau.
"Contrexéville." By Dr. Debout d' Estrées.

Albert Broadbent, Manchester.
"The Building of the Body."

Thompson and Jamieson.
"A Few Words of Advice to Nurses." By Esther H. Young.

Blackie and Son, Ltd.
"Elementary Ophthalmic Optics." By Freeland Fergus.

W. H. and L. Collingridge.
"The City of London Directory, 1903."

### SCRAPS AND GLEANINGS.

In six years nearly 450,000 players have visited the municipal golf course at Edinburgh.

The Clothworkers' Company have sent a donation of £50 to the Royal Association In Aid of the Deaf and Dumb.

Telephonic communication took place between Vienna and Hamburg for the first time on Saturday, April 25th.

Two more cases of small-pox are reported as having occurred at Kingston-on-Thames, making 12 since the outbreak a few weeks ago.

The largest steam-tug ever built has just been launched from a German shipyard. She is 168 feet long, can develop 1,500-horse power, and is fitted with wireless telegraphy.

The recent fall of snow in Austria has been so heavy that during the funeral of an Austrian bishop the snow had continually to be dug up to enable the procession to reach its destination.

It is stated that a large number of people, of nearly every class of society have, at the instance of the National Association for the Suppression of Bad Language, signed the pledge against bad language.

Four blocks of stone, which are said to have originally formed part of the Emperor Hadrian's Roman wall, are to be preserved on board the new Cunarder *Carpathia*. They were dug up in the shipyard at Wallsend.

At the recent annual meeting of the London Adult School Union, it was stated that 43 men's and 17 women's schools were in association, and that during the past year the average attendance had increased by 50 per cent.

### THE STUDENT OF MEDICINE.

### APPOINTMENTS.

**Reginald Alderson**, M.D., B.S.Durh., appointed Honorary Assistant Surgeon to the Hospital for Sick Children, Newcastle-upon-Tyne. **C. H. Auty**, M.R.C.S.Eng., L.R.C.P.Lond., appointed District Medical Officer of the Parish of Willesden. **F. G. Bennett**, L.S.A., appointed District Medical Officer to the Billesdon Union. **George Byres**, M.B., M.S.Aberd., appointed Medical Officer for the Parish of Foveran, and Surgeon to H.M. Coastguards. **Katherine Chamberlain**, M.B., B.S.Lond., appointed Resident Physician to the Royal Free Hospital, Gray's Inn Road. **Thomas Easton** M.D.Edin., C.M., appointed District Medical Officer of the Southampton Union. **T. A. Green**, M.D., C.M., appointed Clinical Assistant to the Chelsea Hospital for Women. **John T. Hewetson**, M.D., F.R.C.S., appointed Assistant Obstetric Officer to the General Hospital, Birmingham. **Sidney Hillier**, M.D.Edin., C.M., appointed Medical Officer to the Children's Home of the Stow Union. **F. C. Jobson**, M.R.C.S., L.R.C.P.Lond., appointed Assistant Medical Officer of the Poplar and Stepney Sick Asylum District. **James Johnson**, L.R.C.P. and S.Edin., L.F.P.S.Glasg., appointed Medical Officer of Health for Bispham and Morbrick, Poulton-le Fylde. **George Knapton**, L.R.C.P.Edin., appointed a Physician to the Edinburgh Life Assurance Company for Manchester, Bowdon, and District. **James Mackay**, M.B.Aberd., appointed a Physician to the Edinburgh Life Assurance Company for Manchester and District. **John Alexander McKenzie**, M.B., Ch.B., appointed Resident Physician to the Aberdeen Royal Infirmary. **H. L. Ormerod**, M.D., R.U.I., B.Ch., appointed Medical Officer for the Westbury District of the Barton Regis Union. **Frederick K. Smith**, M.B., Ch.B., appointed Resident Surgeon to the Aberdeen Royal Infirmary. **W. Langford Symes**, M.D., Univ. Durh., F.R.C.P.I., appointed Pathologist to the Royal City of Dublin Hospital. **A. J. Thomson**, M.R.C.S., L.R.C.P.Lond., appointed Medical Officer of the Stourbridge Union Workhouse. **Edward Paget Thurstan**, M.D., B.A.Cantab., M.R.C.S.Eng., L.S.A.Lond., appointed Physician to the Perth Public Hospital. **Herbert Tilley**, F.R.C.S., M.D., appointed Examiner in Laryngology, Royal Army Medical College. **T. E. Frazer Toovey**, F.R.C.S., L.R.C.P., appointed House Surgeon to the Royal Eye Hospital, Southwark. **Noel N. Wade**, M.B., Ch.B.Edin., appointed Assistant House Surgeon to the Cardiff Infirmary. **I. S. Warrack**, M.A., M.D.Aberd., D.P.H.Camb., Boarding Medical Officer to Port of London Sanitary Authority, Gravesend.

### "The Hospital" Institutional Library.

"Hospitals and Asylums of the World." 4 Vols., with a Portfolio of Plans, complete £12 12s.

"Burdett's Hospitals and Charities, 1902." 5s.

"Burdett's Official Nursing Directory, 1903." 3s. net.

"Cottage Hospitals, General, Fever, and Convalescent." 10s. 6d.

"Uniform System of Accounts for Hospitals and Public Institutions." New Edition. 4s. net.

"Hospital Expenditure: The Commissariat." 2s. 6d.

"A Legal Handbook for the Use of Hospital Authorities." 2s. 6d.

"The Cottage Hospital Case Book or Register of Patients." 10s. and 12s. 6d.

"The Furnishing and Appliances of a Cottage." 6d.

All these are published by the Scientific Press, Ltd., and may be obtained through any bookseller, or direct from the publishers, 28 and 29 Southampton Street, Strand, London, W.C.

### Medical Appointments.

MEDICAL DEPARTMENT OF THE NAVY,
ADMIRALTY, Northumberland Avenue, W.C.
10th February, 1903.

An EXAMINATION of CANDIDATES for entry into the Medical Department of the Royal Navy will be held on the 11th MAY next and following days at Examination Hall, Thames Embankment.

The forms to be filled up by candidates will be supplied on application to this Department.

H. F. NORBURY,
Director-General.
(38)

ABERDEEN ROYAL INFIRMARY.

### Hospital Matrons,

Do you know the best book on how to provide for the Hospital Household? In

### HOSPITAL EXPENDITURE: The Commissariat,

Price **2/6** post free, you will find every question considered which bears upon this important subject. The varying circumstances of large and small institutions are carefully considered, and from the making of beef-tea to the complete cost of diets everything that can interest and assist an intelligent manager is within its pages.

You can compare your methods with those of other Hospitals, because careful comparisons are made, even in the matter of the sale of waste material. Matrons about to occupy new posts will find this book invaluable, as it will place them at once in that position of power which knowledge gives.

### The Cottage Hospital Matron will find useful

### COTTAGE HOSPITALS,

**By Sir HENRY BURDETT,**

Price **10/6** post free, 3rd Edition, and a little pamphlet on the

### FURNISHING AND APPLIANCES OF A COTTAGE HOSPITAL,

**By the Hon. SYDNEY HOLLAND,**

Price **6d.** post free; and those matrons who have no secretarial assistance, and all secretaries and medical officers will find invaluable

### THE COTTAGE HOSPITAL CASE-BOOK,

**Or, REGISTER OF PATIENTS.**

Price **10/-** post free.

It is arranged for 150 in-patient cases, with rulings for 8 weeks' payments to each case.

Larger size, containing space for about 300 in-patient cases, **12/6** post free.

**All these books can be obtained at all Booksellers', and at**

**THE SCIENTIFIC PRESS, 28 Southampton Street, Strand, W.C.**

